# Awake sigmoidectomy under continuous spinal anesthesia in a high-risk ASA class V patient: a case report

**DOI:** 10.1186/s13037-025-00468-6

**Published:** 2025-12-20

**Authors:** Henry Abumohor, Yara Taha, Seham Madaka, Majdi Hmamdeh, Ahmad Irzeiqat, Mohammed Maraqa

**Affiliations:** 1 Faculty of Medicine, Medical Research Club, Al-Quds University, Jerusalem, Palestine; 2 Department of Anesthesiology, AL-Ahli Hospital, Hebron, Palestine; 3 Department of Surgery, AL-Ahli Hospital, Hebron, Palestine

**Keywords:** Awake surgery, Continuous segmental spinal anesthesia, Regional anesthesia, Sigmoidectomy

## Abstract

**Background:**

Awake colorectal surgery is an alternative to general anesthesia, especially in high-risk patients with significant cardiopulmonary comorbidities. Continuous segmental spinal anesthesia offers stable intraoperative conditions while avoiding the complications of cardio depressant effects, airway manipulation and sedation. Despite its advantages, this approach remains underreported in major colorectal procedures.

**Case presentation:**

We present the case of a 73-year-old male with a history of ischemic cardiomyopathy, chronic heart failure (ejection fraction 25–30%), chronic kidney disease, and pulmonary complications, classified as American Society of Anesthesiologists (ASA) Risk Class V. The patient was admitted to a tertiary hospital in Palestine with a recurrent sigmoid volvulus, and due to the high risk associated with general anesthesia, the surgical, cardiology and anesthesia teams decided for an awake open sigmoidectomy under continuous segmental spinal anesthesia. The patient remained fully conscious and hemodynamically stable throughout the procedure. Postoperative recovery was uncomplicated apart from a superficial wound infection, which was managed conservatively. At six-month follow-up, the patient remained asymptomatic, tolerated a normal diet, had no recurrent bowel obstruction, and showed complete wound healing without late infectious or anastomotic complications.

**Conclusion:**

This case illustrates the feasibility, safety, and potential benefits of awake sigmoidectomy in fragile, multimorbid patients. To our knowledge, it represents the first reported case in Palestine. The successful outcome emphasizes the importance of advanced regional techniques and multidisciplinary collaboration in managing high-risk surgical patients, especially in settings with limited resources.

## Background

Awake surgery is the term used to describe surgical procedures performed under regional anesthesia while the patient is still conscious and breathing on their own, without the need for general anesthesia or endotracheal intubation [1]. This approach has become increasingly popular in recent years, particularly for patients with significant cardiopulmonary comorbidities, for whom general anesthesia carries a significant risk. Several specialties have seen success with awake techniques, including urology, orthopedics, gynecology, and increasing abdominal surgery. This expansion reflects growing evidence of the benefits of regional anesthesia in minimizing perioperative risks, particularly in gastrointestinal procedures [2, 3].

Although regional anesthesia has been reported for some laparoscopic procedures, the available evidence remains limited. In the context of gastrointestinal procedures such as liver resection, hernia repair, laparoscopic cholecystectomy, and colectomy, regional anesthesia has demonstrated promise. These methods support quicker postoperative recovery and reduce the risks of airway manipulation and cardio depressant effects particularly in elderly or fragile patients [4, 5].

Reports clearly detailing awake colorectal procedures, like sigmoidectomy, are still limited, even though applications in gastrointestinal surgery are expanding. Despite these advantages, its use remains limited, partly due to technical challenges such as catheter placement, precise dosing, and the need for specialized expertise. There are currently no official guidelines, and the majority of the evidence is derived from case reports or small series [6].

A recent report from Palestine described a case where a high-risk patient successfully underwent sigmoidectomy and colostomy using thoracic combined spinal-epidural anesthesia. The patient presented with comorbid chronic kidney disease, heart failure, ischemic heart disease, and pulmonary edema. In older adults, general anesthesia can inhibit the smooth muscle of the heart and arteries, leading to unpredictable physiological reactions and a decreased ability to withstand the stress of surgery. To reduce these risks, the multidisciplinary team chose this form of anesthesia for better control of complex clinical conditions [7].

In this report, we describe the successful awake open sigmoidectomy for recurrent sigmoid volvulus in a high-risk elderly male patient with ischemic cardiomyopathy and a significantly reduced ejection fraction, under continuous segmental spinal anesthesia. According to, the patient has American Society of Anesthesiologists (ASA) Risk Class V, which indicates that the patient is moribund and unlikely to survive without the procedure. To our knowledge, this is the first reported case in Palestine of an awake sigmoidectomy using continuous segmental spinal anesthesia in a high-risk patient, demonstrating the feasibility and safety of this approach.

## Case presentation

### Patient profile

A 73-year-old male presented to the emergency department of a tertiary hospital in Palestine, with acute abdominal pain, vomiting, and abdominal distention. On admission, he denied any chest pain and he was hemodynamically stable on arrival. His past medical history was significant for coronary artery disease, chronic heart failure with a reduced ejection fraction of 25–30%, hypertension, diabetes mellitus, and chronic kidney disease. He was classified as ASA Risk Class V He had undergone coronary artery bypass grafting four years earlier. At the time of the presentation, he was admitted to the cardiac care unit. ECG and troponin testing showed no signs of acute ischemia or myocardial infarction, and echocardiography confirmed severely reduced left ventricular function.

### Diagnostic assessments

Standing abdominal X-ray revealed signs of large bowel obstruction due to sigmoid volvulus. On the first day of admission, the patient underwent urgent endoscopic decompression with rectal tube placement. He initially improved, and on the third day of admission, the rectal tube was removed. But by the fifth day of admission, symptoms recurred, and a standing abdominal X-ray confirmed recurrent sigmoid volvulus. The rectal tube was reinserted, with temporary improvement. On the seventh day of admission, upper and lower GI endoscopies were performed. Gastroscopy revealed mild esophagitis, aphthous ulcers in the duodenal bulb, and minimal gastritis, while colonoscopy showed no abnormalities throughout the colon and terminal ileum.

### Surgical intervention

Given the recurrence of volvulus and failure of conservative management, surgical intervention was deemed necessary. However, the patient’s severely impaired cardiac function made general anesthesia a high-risk option. A multidisciplinary team consisting of a surgeon, anesthesiologist, and cardiologist carefully evaluated the case and elected to proceed with surgery under continuous segmental spinal anesthesia.

On the eighth day of admission, the patient underwent an urgent open sigmoidectomy in the supine position. Spinal anesthesia was administered via the T11–T12 space using an 18-G Tuohy needle inserted to a depth of 5.5 cm, and a catheter was advanced to 9 cm. An initial bolus of 7.5 mg of 0.5% Bupivacaine isobaric was given, followed by a continuous infusion of 0.125% Bupivacaine isobaric at 1–2 ml/hour. Local infiltration with 1% lidocaine was used for skin anesthesia. The anesthesia induction began, with continuous spinal anesthesia initiated after one hour and a half. Throughout the procedure, the patient remained stable, with systolic blood pressure ranging from 130 to 140 mmHg, diastolic pressure from 60 to 70 mmHg, and oxygen saturation maintained at 99% on spontaneous ventilation.

After surgical prepping, Foley’s catheter and NGT were inserted. A lower midline incision was made. Intraoperative exploration revealed a markedly redundant sigmoid colon measuring approximately 20 cm. A mesenteric window was created using a LigaSure device, and the mesentery was ligated and divided. The redundant sigmoid segment was resected. Proximal and distal bowel ends were opened longitudinally, and a side-to-side colorectal anastomosis was performed using a GIA stapler. The staple line was reinforced with interrupted 2 − 0 Vicryl sutures, followed by a second layer of seromuscular sutures. The mesenteric window was closed, and hemostasis was secured. An abdominal drain was placed near the anastomosis site, and a rectal tube was inserted and fixed. These interventions reflect standard practice in high-risk colorectal surgery at our institution and were not prompted by any intraoperative complication. The abdomen was closed in layers, and the wound was dressed. The procedure was completed without intraoperative complications.

Preoperative medications included intravenous metronidazole 500 mg, ceftriaxone 1 g, and atropine 0.6 mg. Intravenous fluid therapy consisted of two 500 mL boluses of Ringer’s lactate and 1,000 mL of crystalloids. Intraoperative urine output was measured at 500 mL, and the estimated blood loss was approximately 200 mL.

### Postoperative management

The patient was transferred to the surgical ICU for postoperative monitoring. On postoperative day two, the patient developed localized erythema and mild tenderness around the lower third of the midline incision. The wound edges remained well-approximated with no dehiscence, and there was a small amount of seropurulent discharge without deep tracking or systemic signs of infection. The diagnosis of a superficial incisional surgical-site infection was made based on clinical examination according to CDC criteria, limited to skin and subcutaneous tissue without involvement of the fascia or muscle layers. There was no fever, leukocytosis, or hemodynamic instability.

The infected portion of the incision was opened at the bedside for drainage, irrigated with normal saline, and left partially open to heal by secondary intention. Daily wound care consisted of saline irrigation and moist-to-dry dressing changes performed by the nursing staff. Initial empiric antibiotics were escalated to meropenem (1 g IV every 8 h) after the wound swab culture revealed Escherichia coli sensitive to carbapenems. The surrounding erythema progressively regressed, and complete resolution of the infection occurred within several days. The wound continued to granulate well, and there were no long-term complications at outpatient follow-up. The rectal tube was removed on the fifteenth day of admission, and the patient showed consistent improvement.

On day 10, a postoperative CT scan with oral and IV contrast revealed expected postoperative changes, including mild pneumoperitoneum, surgical emphysema at the midline abdominal wall, and the presence of an abdominal drain. There was diffuse haziness of the mesentery, mild free fluid, and mild reactive wall thickening of small bowel loops.

Mild cardiomegaly, bilateral pleural effusions, emphysematous and bronchiectatic changes, and a 7 mm subpleural nodule in the right middle lobe were observed on CT imaging. Although the pneumoperitoneum had not completely resolved by day 10, this can still be physiological and does not necessarily indicate a complication. A small, contained anastomotic leak was considered as part of the differential; however, the intraperitoneal drain showed only minimal serous output without feculent material, blood, or increased volume, and there were no biochemical or clinical features suggestive of leakage. The patient remained afebrile and hemodynamically stable, supporting the conclusion that the anastomosis was intact and that no leak was present. Postoperative cardiogram assessment was unchanged from his preoperative status. Incidental findings on imaging were noted but assessed as benign and clinically insignificant.

### Follow-up and outcomes

By the twenty-first day of admission, the patient was afebrile, tolerating oral intake, mobilizing independently, and hemodynamically stable. He was discharged in good condition with instructions to complete his antibiotic course and follow up with general surgery and cardiology. His discharge medications included meropenem 1 g IV three times daily for 7 days, aspirin 100 mg once daily, clopidogrel 75 mg once daily, bisoprolol 5 mg once daily, rosuvastatin 20 mg once daily, amlodipine 5 mg once daily, spironolactone 25 mg once daily, and valsartan/hydrochlorothiazide once daily. The patient was re-evaluated in the surgical outpatient clinic at 1 month, 3 months, and 6 months after discharge. At each visit, he reported normal bowel function, stable cardiopulmonary status, and full return to baseline activities. Physical examination showed a well-healed incision with no evidence of hernia, infection, or anastomotic complications. He experienced no recurrent abdominal pain or obstructive symptoms, and no readmissions occurred during the follow-up period.

### Timeline

**Table:** 

Day of Admission	Event
Day 1	Admission with abdominal pain and vomiting; urgent endoscopic decompression with rectal tube insertion.
Day 3	Clinical improvement noted; rectal tube removed.
Day 5	Recurrence of symptoms; standing abdominal X-ray confirmed recurrent sigmoid volvulus; rectal tube reinserted.
Day 7	Upper GI endoscopy performed (showed mild esophagitis and aphthous ulcers); colonoscopy performed (normal findings).
Day 8	Awake sigmoidectomy performed under continuous spinal anesthesia.
Day 10	Development of superficial wound infection; cultures obtained. Postoperative CT with oral and IV contrast was done.
Day 15	Rectal tube removed after clinical improvement.
Day 21	Patient discharged in stable condition.

## Discussion

In this report, we describe a case of successful awake sigmoidectomy surgery on a 73-year-old male under continuous spinal anesthesia rather than general anesthesia to minimize perioperative risks associated with his multiple cardiac morbidities represented by coronary artery disease, dilated cardiomyopathy with severely impaired left ventricular systolic function (ejection fraction of 25%).

The choice of anesthesia plays an important role in controlling and maintaining perioperative outcomes in patients undergoing high-risk abdominal surgeries, such as sigmoidectomies. Investigations have shown the safety and efficacy of spinal anesthesia in comparison with general anesthesia in patients undergoing abdominal and laparoscopic procedures [8]. Studies have found that patients receiving the combined regional anesthesia experienced a more stable intraoperative and postoperative procedure, reduced opioid consumption, longer postoperative analgesia with fewer side effects, and higher patient and surgeon satisfaction compared to those who underwent procedures under general anesthesia. This technique of avoiding general anesthesia and the usage of low dose spinal block combined with epidural was proven to be more effective in patients with poor left ventricular function. This approach may be beneficial for patients undergoing surgical procedures but are at high risk for hemodynamic instability [9].

Regional anesthesia is emerging as a safe and effective alternative to general anesthesia, especially in high-risk patients suffering from cardiopulmonary diseases who are undergoing major surgeries. A study on patients suffering from congestive heart failure undergoing lower extremity amputation, found that patients who underwent the surgery under regional anesthesia experienced significantly lower rates of pneumonia, septic shock, and 30-day mortality compared with those who received general anesthesia. Additionally, geriatric patients undergoing lower extremity revascularization were examined for the type of anesthesia they received and their postoperative outcomes. The study revealed that patients undergoing surgery under regional anesthesia experienced decreased short- and long-term mortality rates. They also needed lower postoperative oxygen therapy and mechanical ventilation [8, 9].

Similarly, a 72-year-old morbidly obese female with multiple comorbidities has benefited from Thoracic Combined Spinal-Epidural Anesthesia when undergoing an open surgical repair for an incarcerated abdominal hernia. The procedure proceeded smoothly without any cardiopulmonary complications, reinforcing the technique’s safety and effectiveness in high-risk patients [10]. In our case, the patient had a history of multiple cardiac morbidities that made him vulnerable to side effects and postoperative complications. The use of awake epidural anesthesia provided effective regional anesthesia while maintaining cardiovascular stability, contributing to a smoother perioperative course and fewer complications.

Continuous Segmental Spinal Anesthesia (CSSA) involves the placement of a spinal catheter into the subarachnoid space, allowing continuous or incremental dosing of local anesthetic. This technique enables precise control over the sensory block level and duration, which is especially beneficial in high-risk patients who require hemodynamic stability [11]. In our case, the catheter was inserted at the T11–T12 interspace using a Tuohy needle, and bupivacaine was delivered at a controlled rate to maintain segmental anesthesia targeting the lower abdomen.

This approach allowed the patient to remain awake and spontaneously ventilate throughout the surgery while avoiding significant drops in blood pressure or respiratory compromise. CSSA is particularly useful in patients with poor cardiac reserve, such as those with ischemic cardiomyopathy or heart failure, as it minimizes sympathetic blockade and permits better hemodynamic control [11, 12].

Despite its benefits, regional anesthesia during major abdominal surgery comes with specific challenges. Having modest control of the tone of the abdominal muscles may make it more difficult to expose the underlying structure surgically. Moreover, intraoperative hypotension secondary to sympathetic blockade remains a problem, especially in fragile patients [3]. Worsened anesthesia coverage increases these risks and makes dosing and targeting more difficult. In this instance, using a continuous infusion technique minimizes those risks by precise control of the anesthetic effect. Furthermore, employing segmental spinal anesthesia minimized the pain while allowing the patient to breathe properly [6].

This case highlights the practical feasibility and clinical benefit of performing awake sigmoidectomy under continuous segmental spinal anesthesia in a high-risk, multimorbid patient. It demonstrates that, with appropriate planning and multidisciplinary coordination, major abdominal surgery can be safely carried out without general anesthesia, even in patients with severely compromised cardiopulmonary status. Given the resource limitations in many hospitals in Palestine, including restricted access to advanced cardiopulmonary monitoring and postoperative critical care, the successful use of continuous segmental spinal anesthesia in this case highlights its practical value as a safe alternative for high-risk patients.

## Conclusion

This case highlights that awake sigmoidectomy under continuous segmental spinal anesthesia (CSSA) can be safely and effectively performed in a fragile patient with severe cardiac disease, where general anesthesia posed unacceptable risks. CSSA may represent a valuable alternative in carefully selected high-risk patients.

Further research is needed to better define patient selection criteria, optimize anesthetic techniques, and evaluate long-term outcomes of awake colorectal procedures. Larger studies and prospective trials will be essential to establish standardized protocols and strengthen the evidence base. With structured training and broader clinical experience, CSSA and related regional anesthesia techniques may play an increasingly important role in the safe management of frail surgical populations worldwide.

## Data Availability

No datasets were generated or analysed during the current study.

## Abbreviations

ASA: American Society of Anesthesiologists
CSSA: Continuous Segmental Spinal Anesthesia
